# The Dual PIM/FLT3 Inhibitor MEN1703 Combines Synergistically With Gilteritinib in FLT3‐ITD‐Mutant Acute Myeloid Leukaemia

**DOI:** 10.1111/jcmm.70235

**Published:** 2024-12-09

**Authors:** Sonia Zicari, Giuseppe Merlino, Alessandro Paoli, Alessio Fiascarelli, Patrizia Tunici, Diego Bisignano, Francesco Belli, Clelia Irrissuto, Simone Talucci, Elena Cirigliano, Maria Luisa Iannitto, Mario Bigioni, Alessandro Bressan, Krzysztof Brzózka, Gabriel Ghiaur, Daniela Bellarosa, Monica Binaschi

**Affiliations:** ^1^ Menarini Group Preclinical and Translational Sciences Pomezia Rome Italy; ^2^ Ryvu Therapeutics Krakow Poland; ^3^ Division of Hematological Malignancies, Department of Oncology, Sidney Kimmel Comprehensive Cancer Center Johns Hopkins University School of Medicine Baltimore Maryland USA

**Keywords:** acute myeloid leukaemia, gilteritinib, MEN1703, PIM/FLT3 kinase inhibitor, targeted therapy

## Abstract

MEN1703 is a first‐in‐class, oral, Type I dual PIM/FMS‐like tyrosine kinase 3 inhibitor (FLT3i) investigated in a Phase I/II DIAMOND‐01 trial in patients with acute myeloid leukaemia (AML). Gilteritinib is a highly potent and selective oral FLT3i approved for the treatment of relapsed/refractory AML with FLT3 mutations. Although gilteritinib showed strong single‐agent activity in FLT3‐mutated AML, the development of gilteritinib resistance limits response durability, indicating the importance of novel combination strategies to improve disease outcome. PIM kinases govern FLT3‐ITD signalling and increased PIM kinase expression is found in samples from AML patients relapsing on FLT3i. Here, we report that the simultaneous inhibition of PIM and FLT3, through the combination of MEN1703 and gilteritinib, can consistently improve the in vitro/in vivo antitumor activity over the single agents, demonstrating the benefit of this combination. Moreover, we demonstrate that resistance to gilteritinib can be circumvented by combining MEN1703 with gilteritinib. MEN1703 interferes with FLT3 upregulation, Mcl‐1 overexpression and PIM kinase signalling, which are all involved in FLT3i resistance. We also show that MEN1703 downregulates stromal cytokines that promote cytokine‐mediated resistance of AML blast cells to FLT3 inhibition. These results demonstrate the importance of the combination approach to overcome microenvironment‐mediated resistance to FLT3 inhibitors.

## 1 Introduction

Acute myeloid leukaemia (AML) is a heterogeneous disease of haematopoietic stem cell malignancies characterised by a variegated panel of mutations that lead to rapid expansion of leukaemic progenitors, ultimately causing bone marrow failure. Among this panel, mutations related to the FMS‐like tyrosine kinase 3 (FLT3) receptor play a prominent role. FLT3, a Class III receptor tyrosine kinase, is highly expressed on the surface of many haematopoietic stem cells [1]. Internal tandem duplication (ITD) mutations in the juxtamembrane domain of FLT3 are found in approximately 25% of AML cases and result in constitutive activation of downstream signalling, causing increased proliferation and survival of myeloid precursor cells [2]. The presence of FLT3‐ITD mutations confers an extremely poor prognosis due to activation of multiple downstream survival pathways, including MAPK/ERK, PI3K/AKT and JAK/STAT [3].

Gilteritinib is a FLT3 inhibitor approved for FLT3‐mutated relapsed/refractory AML. Compared to chemotherapy, gilteritinib improves overall survival in this population [4]. Unfortunately, gilteritinib monotherapy has resulted in only short‐term responses due to the emergence of resistance mediated by bone microenvironment (early, non‐mutational) and by later expansion of mutated clones, highlighting the need for combination strategies to prevent resistance [5]. Approaches combining FLT3 inhibitors with other targeted therapies have been explored, with some under investigation [6, 7, 8].

PIM (proviral integration site for Moloney murine leukaemia virus) kinases, comprising three highly homologous serine/threonine kinases, are involved in the pathogenesis and progression of multiple malignancies, including AML and are important effectors of FLT3‐ITD activity. PIM can phosphorylate FLT3 in a positive feedback loop and act as a major driver of the FLT3‐resistance phenotype [9, 10, 11]. PIM overexpression has been well documented as a secondary, non‐mutational resistance mechanism, resulting in FLT3‐ITD upregulation and increased expression of apoptotic factors, such as bcl‐2, bcl‐xl and Mcl‐1 [12].

MEN1703/SEL24 (MEN1703) is a first‐in‐class, oral, Type I dual PIM/FLT3 inhibitor that has been investigated as a single agent in a Phase 2 study in AML (DIAMOND‐01, NCT03008187). MEN1703 has received FDA orphan drug designation for AML. The on‐target activity of MEN1703 on PIM‐phosphorylated substrates has been well characterised and inhibition of downstream modulators such as pS6 has been used as pharmacodynamic biomarkers in clinical trials [9, 13]. In this study, we investigated whether the combination of MEN1703 with gilteritinib in in vitro and in vivo preclinical FLT3‐ITD AML models could improve antitumor activity over single agents.

## 2 Materials and Methods

### 2.1 Drugs and Reagents

Details on drugs and reagents are in the [Link], [Link], [Link].

### 2.2 Human Samples

Specimens were collected by Normal and Oncological Tissue Collection Hub (NOTCH) at Sidney Kimmel Comprehensive Cancer Center. Informed consent was obtained from all donors before specimen collection, in accordance with the Declaration of Helsinki and under a research protocol approved by the Johns Hopkins Institutional Review Board. Human bone marrow stroma cells were derived from healthy volunteers as previously described [14]. Blasts were isolated from AML patients with FLT3 mutations.

### 2.3 Cell Lines

Human AML cell lines FLT3‐ITD MOLM‐13 and MOLM‐14 (DSMZ, Braunschweig, Germany) were cultured in RPMI 1640 medium (Gibco, Life Technologies, Carlsbad, CA, USA) supplemented with 20% fetal bovine serum (FBS) (Sigma). MV‐4‐11 (ATCC) cell lines were grown in RPMI10% FBS. All cells were incubated at 37°C, 5% CO_2_ and 85%–90% relative humidity.

### 2.4 Ex Vivo Assay

To test the potential synergy between MEN1703 and gilteritinib in AML cell lines in the presence of stroma, primary human BMSCs were plated in 96‐well plates; when monolayer was confirmed, luciferase‐expressing AML cells (MV‐4‐11 or MOLM‐14) were layered on top and treated with gilteritinib, MEN1703 or their combination. Cell viability was measured after 72 h using bioluminescence. Drug effect was determined relative to untreated controls. The combination index (CI) was calculated using the ratio of expected effect/observed effect.

To test if MEN1703 and gilteritinib have synergistic effects on FLT3‐mutant AML ex vivo, blasts were isolated from AML patients with FLT3 mutations that were gilteritinib‐naïve or ‐resistant. We determined the IC_25_/IC_50_/IC_75_, then treated primary blasts with MEN1703, gilteritinib or their combination. The drug effect was measured using CellTiter 96 Aqueous One Solution Reagent (MTS) (Promega, Madison, WI, USA) assay, according to manufacturer's instructions, at 72 h post‐treatment. CI was calculated as above.

### 2.5 Cell Viability Assay and In Vitro Gilteritinib‐Resistant Model

Cells were seeded in 96‐well plates at 50,000 cells per well prior to addition of MEN1703 or gilteritinib at Day 0, with or without cytokines (20 ng/mL). The concentrations used for MEN1703 ranged from 20 μM to 9.76 nM with a twofold dilution. The concentrations used for gilteritinib ranged from 6 μM to 0.09 nM with a threefold dilution. All experiments were performed in triplicate.

The concentration range was then used for the combination of MEN1703 plus gilteritinib to include IC_10_/IC_25_/IC_50_/IC_75_ values for each drug and cell line. After 72 h, MTS was added to assess cell viability. Fluorescence was measured 2–4 h later using Tecan Infinite M200 (Tecan Trading AG, Switzerland), recording absorbance at 490 nm.

Quantitative measurement of synergism/antagonism was evaluated through CompuSyn (ComboSyn Inc., Paramus, NJ, USA), with analysis of the CI. CI < 1, = 1 and > 1, indicates synergistic, additive and antagonistic effects, respectively. The fraction affected (Fa) indicates the fraction of cell viability affected by the combination of drugs, with higher Fa (on a scale from 0 to 1) indicating higher cell mortality (effect) induced by the combination. Combenefit platform d (Cancer Research UK Cambridge Institute) was used to map combination effect as dose–response surface according to Loewe model to D‐R (LOEWE).

### 2.6 Colony‐Forming Assay

MV‐4‐11 cells were treated (or not) with different concentrations of MEN1703 (62.5–125 and 250 nM), gilteritinib (2.5–5 and 10 nM) or their combination. After 72 h, cells were collected and plated (200/well) in methylcellulose medium (composed by Methocult H4230 [STEMCELL Technologies, Vancouver, BC, Canada] + RMPI 10% FBS). Plates were maintained at 37°C, 5% CO_2_ and 85%–90% relative humidity. After 10 days, colonies per well were counted.

### 2.7 RNA‐Seq Analysis

RNA was extracted from 5 × 10^6^ MV‐4‐11 cells, treated (or not) for 24 h with 200 nM MEN1703, 5 nM gilteritinib or their combination, using the RNeasy Mini kit (250), following manufacturer's instructions (Qiagen, Hilden, Germany). RNA‐Seq libraries were quantified using Qubit 4 Fluorometer with the Qubit 1X dsDNA High Sensitivity (HS) Assay Kit and 4200 TapeStation System with High Sensitivity D1000 Screentape and High Sensitivity D1000 Reagents (Thermo Fisher Scientific; see [Link], [Link], [Link]). Libraries were pooled and cloned onto sequencing spheres using an Ion Chef system. Templated spheres were loaded on Ion 540 chips and sequenced on an Ion Torrent S5 DNA sequencer (Thermo Fisher Scientific). Data were aligned to the hg19 reference genome using CLC Workbench 22.0.2 (Qiagen).

### 2.8 Differential Expression Analysis

RNA‐Seq data were analysed using CLC Genomics Workbench (Qiagen Bioinformatics) and mapped with the following parameters: (a) maximum number of allowed mismatches: 2; (b) minimum length and similarity fraction: 0.8; and (c) minimum number of hits per read: 10. Gene expression values were reported as TPM (transcript per million mapped reads). The threshold *p* value was determined according to the false discovery rate (FDR). Genes that were identified as being differentially expressed met the following criteria: *p* ≤ 0.05 and absolute fold change of 1.5.

### 2.9 Gene Set Enrichment and Modelling of Gene Interaction Networks

Differentially expressed genes were imported into Ingenuity Pathways Analysis (IPA) software and subjected to functional annotations and regulatory network analysis using canonical pathway analysis and upstream regulator analysis. The *p* value (negative log of P) represents the possibility of genes in the network being found together by chance. The activation *z* score predicts the activation state of the upstream regulator, using the molecule expression patterns of molecules downstream of an upstream regulator. An absolute *z* score of ≥ 2 is considered significant.

### 2.10 Capillary Electrophoresis Immunodetection

Cell lysates were prepared using the radioimmunoprecipitation (RIPA) buffer (Thermo Fisher Scientific) supplemented with cOmplete Mini protease inhibitors (Roche) and PhosSTOP phosphatase inhibitors (Roche). Proteins of interest were analysed by JESS (ProteinSimple Inc.) capillary electrophoresis‐based immunodetection system. Peak area calculations were performed by Compass Software, using the Gaussian method for peak fitting. Peak areas of the target proteins were normalised to the peak area of the corresponding total protein, for phosphorylated target or to the peak area of GAPDH or actin, for the other proteins and reported as a percentage of the control. All experiments were performed in triplicate and mean ± SD is shown as a bar plot.

### 2.11 In Vivo Xenograft Models

For the AML xenograft models, 10 × 10^6^ MV‐4‐11 or MOLM‐13 cells were resuspended in 0.2 mL of BME type III (Trevigen) at 5.6 mg/mL and injected subcutaneously into the right flank of 6–8‐week‐old female SCID mice (Charles River, Calco, Italy). Mice were maintained in micro isolator cages under continuously monitored environmental conditions. Drinking water and a specific sterilised diet (VRF1, Charles River) were supplied ad libitum. Environmental conditions and procedures for animal housing and handling complied with the UKCCCR guideline and the European Convention for the protection of vertebrate animals used for experimental and other scientific purposes [15, 16]. Twice weekly, tumour growth and body weight were recorded. For outcome evaluation, tumour volumes were measured by calliper and tumour masses were calculated using the following formula: [length (mm) × width 2 (mm) × *d*/2], assuming density, *d* = 1 mg/mm^3^ for tumour tissue [17].

When the average tumour volume reached 200–300 mm^3^, animals were randomly assigned into four groups (6–7 mice/group). Group I received vehicle (gilteritinib diluent solution) carboxymethyl cellulose 0.5% orally q1xd22 or q1xd21. Group II received MEN1703 at 25 mg/kg orally q1dx14. Group III received gilteritinib at 30 mg/kg orally q1xd21 or at 3 mg/kg q1xd22. Group IV received the doublet combination of MEN1703 + gilteritinib.

Treatment effectiveness was assessed as tumour volume inhibition (TVI) percentage in treated versus control mice, using the following formula:

$$\mathrm{TVI}\%=\left(1-\text{Volume Average of treated tumor mass}/\text{Volume Average of control tumor}\right)\times 100.$$

Mice were sacrificed when tumours reached a volume of approximately 10% of total body weight or when mice's body weight decreased by > 20% compared to control animals for ≥ 7 days. Animals were euthanised via carbon dioxide exposure according to standard procedures [16].

For each treatment group, three mice were sacrificed after the first four doses of the drugs to perform biomarker analysis on the tumour masses.

### 2.12 Efficacy Studies in Patient‐Derived Xenograft Model CTG–2228

The AML diffuse patient‐derived xenograft model (PDX) model CTG‐2228, developed by intravenously inoculating human AML blasts from patients into immunocompromised mice, was performed by Champions Oncology. A surrogate cohort of animals (*n* = 3–5) was sacrificed for AML burden analysis in the bone marrow by fluorescence‐activated cell sorting (FACS) at various time points prior to the estimated engraftment window. Once surrogate animals had sufficient engraftment levels in the bone marrow (% human CD45+ (hCD45) of live cells ≥ 20% average), the remaining pre‐study animals were randomised based on body weight and assigned to four groups. Group I received vehicle (gilteritinib diluent solution) carboxymethyl cellulose 0.5% q1xd21; Group II received MEN1703 at 15 mg/kg per orally q1dx14; Group III received gilteritinib at 15 mg/kg orally q1xd21; and Group IV received the combination of MEN1703 + gilteritinib.

To follow the tumour burden in peripheral blood over time, mice were sampled via submandibular bleed on Days 0, 6 and 13 and analysed by FACS for hCD45 and hCD33 markers. All mice were sacrificed at the end of the study (Day 48) to perform FACS analysis on bone marrow and spleen (additional details in [Link], [Link], [Link]). The tumour burdens in peripheral blood, bone marrow and spleen were evaluated via FACS analysis.

### 2.13 Statistical Analysis

GraphPad Prism software, v. 9.3.1 (GraphPAD Software Inc., CA) was used for statistical analysis. For in vitro studies, statistical differences were considered significant at *p* < 0.05 using two‐way ANOVA (Dunnett's multiple comparison test) for cytokine experiments and one‐way ANOVA for CFU assay.

For in vivo studies, statistical differences were considered significant at *p* < 0.05 using one‐way ANOVA or two‐tailed Mann–Whitney rank test.

## 3 Results

### 3.1 MEN1703 and Gilteritinib Differentially Modulate Phosphorylated and Total FLT3

**FIGURE 1:**
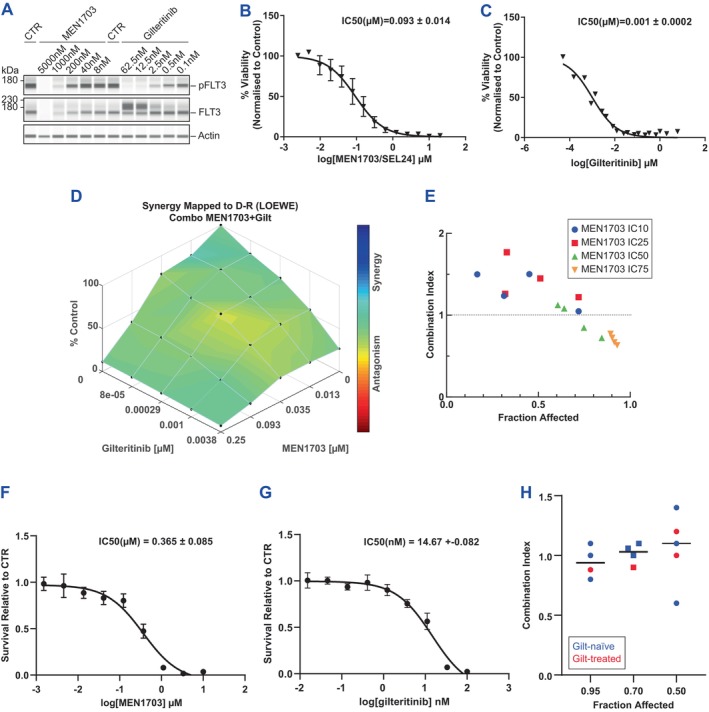
MEN1703 inhibits the FLT3 pathway differently from gilteritinib and in vitro combination with gilteritinib determines a synergistic drug interaction in FLT3‐ITD AML cells. (A) The FLT3 pathway was analysed in the MV‐4‐11 cell line. Cells were treated for 24 h with MEN1703 or gilteritinib and vehicle control (CTR), and pFLT3 (Y589‐591) and total FLT3 levels were determined by capillary electrophoresis immunodetection. Representative picture of three independent experiments. (B) Cytotoxicity of MEN1703 was assessed in MV‐4‐11 cells by MTS assay after 72 h of treatment with drugs. (C) Cytotoxicity of gilteritinib was assessed in MV‐4‐11 cells by MTS assay after 72 h of treatment with drugs. (D, E) Combined MEN1703 and gilteritinib cytotoxicity was determined in a standard cytotoxicity assay at concentrations indicated in the Materials and Methods. Drug interaction analysis was performed using both Combenefit (D) and Compusyn platforms (E). (F, G) Cytotoxicity of MEN1703 (F) and gilteritinib (G) was assessed in AML primary samples (*n* = 5) from gilteritinib‐naïve and gilteritinib‐treated patients at the indicated concentrations.(H) The MEN1703 and gilteritinib combination index was determined by CompuSyn software.

Because MEN1703 is a potent pan‐PIM inhibitor and can also bind mutated FLT3 [9], we investigated the effect of MEN1703 on phosphorylated and total FLT3 protein compared to gilteritinib. FLT3 modulation was evaluated using immunocapillary electrophoresis to assess the effects of MEN1703 or gilteritinib at a range of concentrations that cover the drug plasma concentrations observed in patients during the DIAMOND‐01 trial (Figure 1A). MEN1703 induced degradation of total FLT3 protein at all tested concentrations; inhibition of FLT3 phosphorylation (pFLT3) was observed only at higher, but clinically relevant, concentrations (Figure 1A and Figure S1). In accordance with previously published literature [18], immunoblot analysis of FLT3 shows two bands corresponding to a simply glycosylated 130 kDa form of FLT3 (p130), which remain in the endoplasmic reticulum (ER) and a complexly glycosylated 160 kDa form (p160) present on the surface. Treatment with gilteritinib resulted in an increased level of p160 isoform, suggesting higher FLT3 cell surface expression. Gilteritinib showed a dose‐dependent inhibition of pFLT3 even at lower concentrations and an upregulation of total FLT3 [19, 20], suggesting that MEN1703 and gilteritinib inhibit FLT3 through a different modality.

### 3.2 In Vitro Combination of MEN1703 With Gilteritinib Results in a Synergistic Drug Interaction in FLT3‐ITD AML Cell Lines

To exploit the dual inhibitory mechanisms of MEN1703, the combination of MEN1703 and gilteritinib was tested in preclinical FLT3‐ITD AML models. In vitro cytotoxicity experiments were performed on three FLT3‐ITD AML cell lines (MV‐4‐11, MOLM‐13 and MOLM‐14). Different levels of inhibitory concentrations of each single drug, determined in cytotoxicity assays (IC_10_/IC_25_/IC_50_/IC_75_) (Figure 1B,C), were combined to obtain a matrix analysed with both Combenefit (Figure 1D) and CompuSyn software (Figure 1E). In MV‐4‐11 cells, the combination demonstrated a mild synergistic effect at higher concentrations. A similar pattern was also observed in MOLM‐13 and MOLM‐14 cells (Figures S2A–D and S2E–H, respectively). These results were confirmed in a colony‐forming unit (CFU) assay in MV‐4‐11 cells, where the combination was synergistic even at lower concentrations (Figure S2I,J). The cytotoxicity of the MEN1703–gilteritinib combination was also explored in AML primary samples obtained from gilteritinib‐naïve and gilteritinib‐treated patients harbouring FLT3‐ITD and other AML‐relevant mutations (Table S1). As shown in Figure 1F–H and in Table S1, the drug interaction between MEN1703 and gilteritinib demonstrated a CI ranging from 0.6 to 1.2, confirming a moderate synergism at higher concentrations.

### 3.3 MEN1703 Downregulates Mcl‐1 and pS6

**FIGURE 2:**
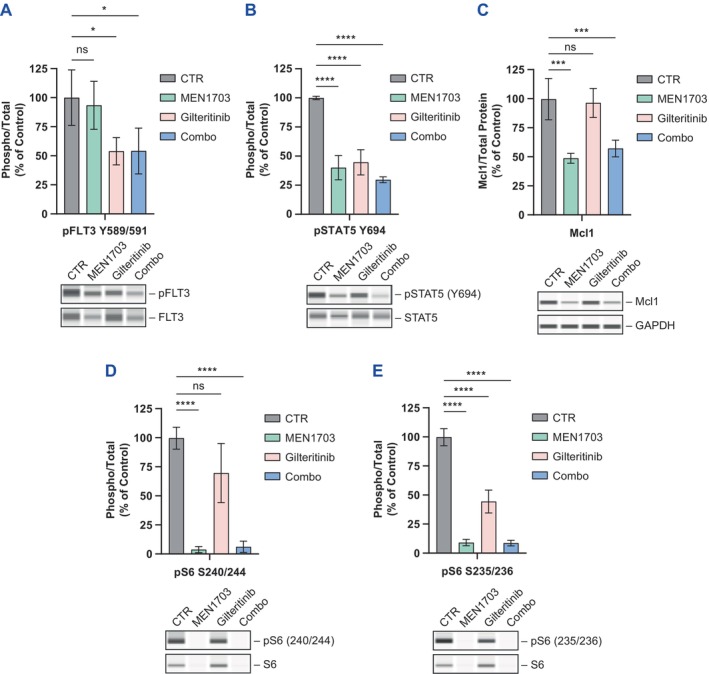
MEN1703 strongly decreases Mcl‐1 and pS6, complementing the gilteritinib mechanism of action in combination treatment. (A–E) Blotting showing biomarker analysis in MV‐4‐11 cells performed by capillary electrophoresis immunodetection after 24 h of treatment at IC_50_ concentrations of the drugs (MEN1703 200 nM, gilteritinib 0.5 nM). (A) pFLT3, (B) pSTAT5, (C) Mcl‐1, (D) pS6 (S240/244) and (E) pS6 (S235/236) were analysed after both single agent and combined treatment. Representative pictures of three independent experiments. *p < 0.05, ***p < 0.001, ****p < 0.0001.

Based on cytotoxicity results, we next sought to investigate the effect of MEN1703 and gilteritinib on biomarkers downstream of PIM and FLT3. The MV‐4‐11 cell line was incubated for 24 h with a concentration of the compounds corresponding to the IC_75_ in the cytotoxicity assay. As previously shown (Figure 1A), pFLT3 inhibition was observed after gilteritinib treatment (50%); a similar decrease was also present in combination with MEN1703. Total FLT3 inhibition mediated by MEN1703 was also observed with the combination treatment, reversing the upregulation induced by gilteritinib (Figure 2A). STAT5 (Y694) phosphorylation was downregulated by both single agents and their combination, consistent with involvement of this biomarker in both PIM and FLT3 signalling (Figure 2B). Indeed, when PIM transcription was analysed, PIM1 mRNA inhibition by MEN1703 and gilteritinib or combination of both drugs showed to be consistent with pFLT3/ pSTAT5 decrease (Figure S3) in accordance with previously published data [21]. In contrast, MEN1703 was able to effectively inhibit Mcl‐1 protein expression (Figure 2C) and S6 phosphorylation (> 90%), both downstream effectors of PIM kinase signalling that were only mildly affected by gilteritinib. MEN1703 also inhibited total S6 levels (Figure 2D,E). The decrease in these biomarkers was maintained with combination treatment at levels comparable to single agents. Treatment with MEN1703 also resulted in cleavage of caspase 3, which was not observed when cells were treated with gilteritinib (Figure S4). The effect was also present with combination treatment. These results suggest cooperation between the respective and complementary mechanisms of action of MEN1703 and gilteritinib.

### 3.4 MEN1703 Inhibits Tumour Microenvironment (TME)‐Related Pathways in a FLT3‐ITD AML Cell Line

**FIGURE 3:**
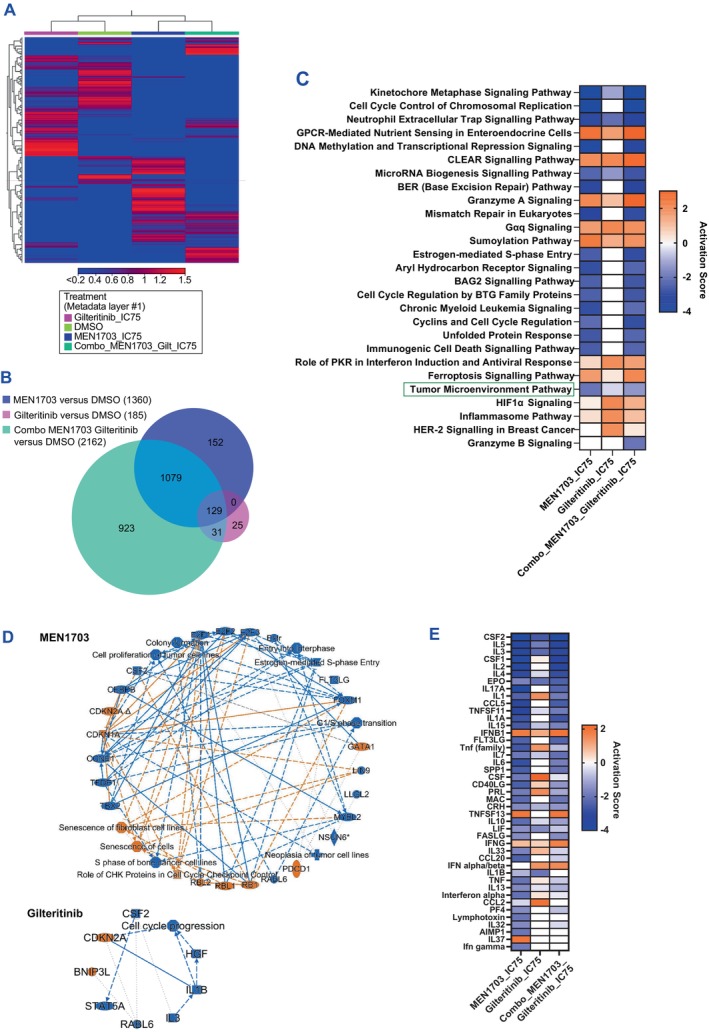
MEN1703 treatment of a FLT3‐ITD AML cell line reveals inhibition of tumour microenvironment pathways. (A) Differentially expressed genes (DEGs) identified by RNA‐Seq analysis between treatments and control sample (DEG defined as *p* < 0.05 and FC > |1.5|). (B) Venn diagram of common and differentially expressed genes (DEGs) between treatments. (C) Canonical pathway analysis of single‐agent and combination treatment (*z* score > 2 and < −2). (D) Network analysis on master regulators and pathways modulated by MEN1703 and gilteritinib. (E) Upstream analysis on cytokines modulated by single‐agent and combination treatment.

To further elucidate the mechanism of action of MEN1703 as a single agent and in combination with gilteritinib, we examined the changes in gene expression upon treatment of MV‐4‐11 cells by RNA‐Seq analysis. Cells were treated for 24 h with the IC_75_ concentrations of MEN1703, gilteritinib or their combination and then processed for RNA extraction. Heatmap analysis (Figure 3A) and Venn diagram (Figure 3B) showed a different pattern of genes modulated by the two compounds and their combination (Table S3 lists all modulated genes); these gene expression patterns were further examined through IPA (Figure 3C,D). Canonical pathway and enrichment analysis revealed that MEN1703 resulted in the most significant enrichment in pathways related to cellular growth and proliferation, DNA methylation, cell cycle regulation, cellular stress and injury and TME pathway (Figure 3C,D). Gilteritinib resulted in a more limited number of enriched pathways, generally related to cell cycle progression and transcriptional regulation (Figure 3C,D). Because the TME pathway affected by MEN1703 is involved in induction of resistance to FLT3 inhibitors [11], we performed an upstream regulator analysis on this pathway through IPA to dissect MEN1703‐modulated factors in more detail. Cytokines involved in AML blast cell survival, remodelling of bone marrow niche and early resistance to gilteritinib (CSF1, IL3, FLT3 ligand, CCL5 and IL6) [22] were significantly downregulated by MEN1703 but not by gilteritinib (Figure 3E).

### 3.5 MEN1703 Impairs the Emergence of Gilteritinib Resistance Induced by Stromal Cytokines

**FIGURE 4:**
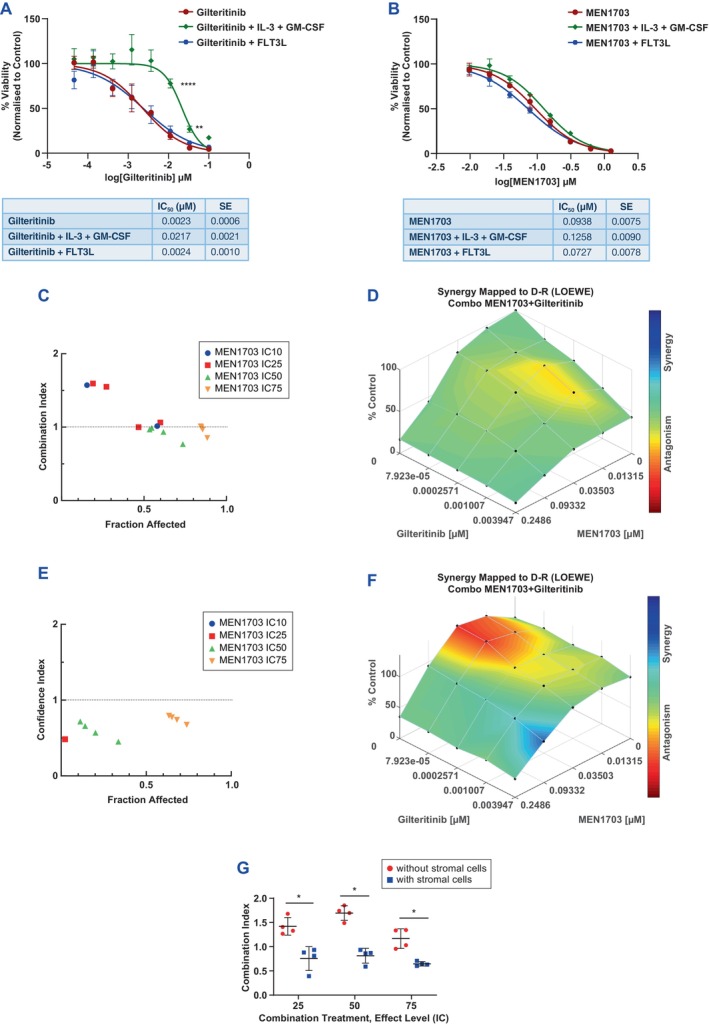
Gilteritinib resistance induced by stromal cytokines in FLT3‐ITD AML cell lines and primary samples is blocked by MEN1703. (A) MV‐4‐11 cells treated with gilteritinib alone or in the presence of IL3 + GM‐CSF or FLT3‐L. Applying two‐way ANOVA (Dunnett's multiple comparison test) between gilteritinib versus gilteritinib + IL3 + GM‐CSF groups, the *p* value at the IC_50_ concentration is statistically significant (*p* < 0.0001). SE = standard error. (B) MV‐4‐11 cells treated with MEN1703 alone or in the presence of IL3 + GM‐CSF or FLT3‐L. Applying two‐way ANOVA (Dunnett's multiple comparison test) between gilteritinib versus gilteritinib + IL3 + GM‐CSF groups, the *p* value at the IC_50_ concentration is not significant (*p* = 0.37). (C, D) Drug‐interaction analysis in the absence of stromal cytokines performed by CompuSyn (C) and Combenefit (D) platforms. (E, F) Drug interaction analysis in the presence of stromal cytokines performed by CompuSyn (E) and Combenefit (F) platforms. (G) Combination index analysis in co‐culture experiments with the MV‐4‐11 cell line with or without primary stromal cells at the dose levels indicated. *p = 0.0286.

Stromal cytokines have been previously shown to contribute to resistance to FLT3 inhibitors [11]. Because RNA‐Seq results suggested involvement of TME factors, we hypothesised that MEN1703 could consistently improve gilteritinib efficacy by counteracting the influence of stromal microenvironment. To test this hypothesis, an in vitro gilteritinib‐refractory model was developed by simultaneously treating MV‐4‐11 cells with gilteritinib (0.046 pM to 100 nM) in the presence of IL‐3/GM‐CSF or FLT3‐L, which are involved in the early phase of gilteritinib resistance (Figure 4A) [11]. Under these conditions, a ninefold increase in the IC_50_ versus gilteritinib alone and to the other stromal factors was observed. In contrast, cytokines/growth factors did not affect MEN1703 activity in a wide range of concentrations (9.76 nM–1.25 μM) in MV‐4‐11 cells (Figure 4B). To determine the effect of selected gilteritinib‐refractory conditions on the combination of MEN1703 plus gilteritinib, we assessed the cytotoxicity of single agents or their combination in the absence (Figure 4C,D) or presence (Figure 4E,F) of IL‐3/GM‐CSF in MV‐4‐11 cells. As shown in Figure 4E,F, the drug interaction became more synergistic when cytokines were added to the cultures. Similar results were obtained with MOLM‐13 and MOLM‐14 cells cultured with G‐CSF (Figure S5A–L, respectively). The ability of MEN1703 to restore sensitivity of gilteritinib‐refractory cells induced by cytokines/growth factors was confirmed in ex vivo co‐culture experiments with MV‐4‐11 and MOLM‐14 cell lines grown in presence of primary stromal cells (Figure 4G and Figure S5M). In MV4‐11 cell line (Figure 4G), the combination index of MEN1703 plus gilteritinib showed a significant decrease in presence of stromal cells; in MOLM‐14 cell line the CI decrease became statistically significant at the IC_75_ dose level (Figure S5M).

### 3.6 Combination of MEN1703 Plus Gilteritinib Potently Inhibits In Vivo Tumour Growth in Xenografted FLT3‐ITD AML Cell Lines and Primary Patient Samples

**FIGURE 5:**
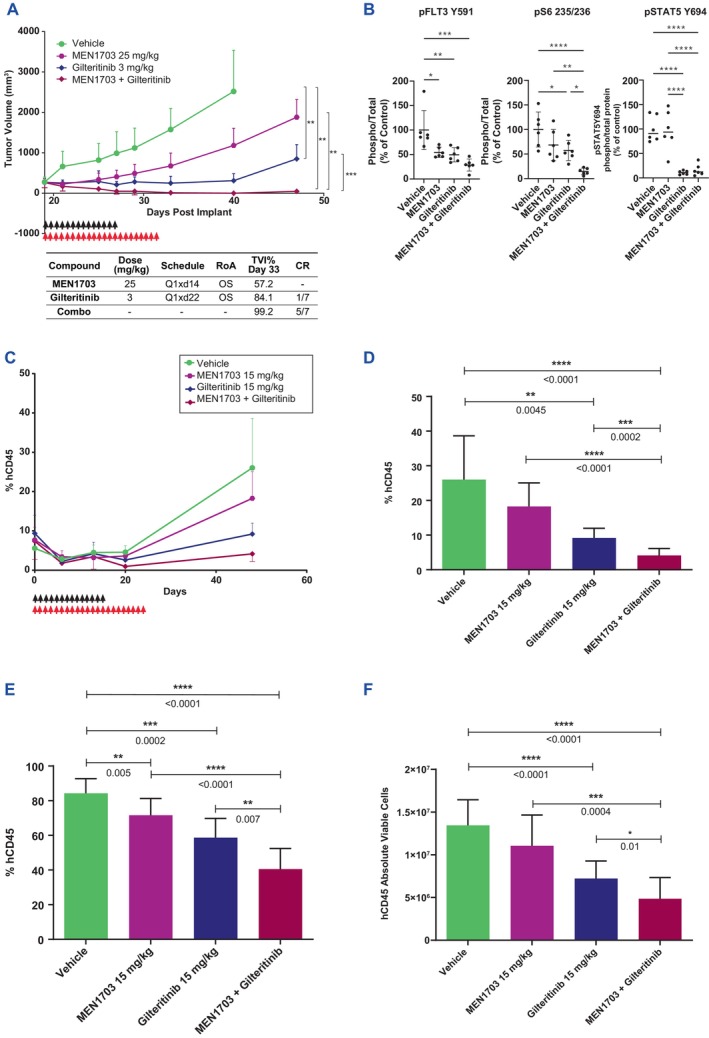
Combination of MEN1703 plus gilteritinib potently inhibits in vivo tumour growth in xenografted FLT3‐ITD AML cell line and primary patient samples. (A) In the MV‐4‐11 xenograft model, mice were treated with 25 mg/kg MEN1703, 3 mg/kg gilteritinib or the combination of both drugs. Statistical analysis was performed at Day 40. Black and red arrows represent MEN1703 and gilteritinib dosing, respectively. TVI = tumour volume inhibition; CR = complete remission. (B) pFLT3, pS6 and pSTAT5 assessment performed by capillary electrophoresis immunodetection in MV‐4‐11 tumour masses treated with single agents and the combination. Pathway inhibition was evaluated by measuring the phosphorylation level of the indicated proteins in tumour nodules after 4 h of treatment. One‐way ANOVA test was applied. (C) Peripheral blood tumour burden of %hCD45 positive cells in the diffuse AML PDX CTG‐2228 model, in which mice were treated with 15 mg/kg MEN1703, 15 mg/kg gilteritinib or the combination of both drugs. (D) Bar graph of peripheral blood tumour burden as %hCD45‐positive cells (Mann–Whitney test was applied at Day 48). (E, F) Bar graph of bone marrow tumour burden as percentage (E) or absolute viable hCD45‐positive cells (F) Mann–Whitney test was applied at Day 48. *p < 0.05, **p < 0.01, ****p < 0.001, ****p < 0.0001.

To confirm that the TME impacts the synergistic interaction between MEN1703 and gilteritinib, we tested both single agents and their combination in a mouse xenograft model developed using MV‐4‐11 cell line engrafted into SCID female mice. In the MV‐4‐11 cell‐derived xenograft model, beginning on Day 16 post‐cell injection, MEN1703 and gilteritinib were administered daily at 25 mg/kg for 14 days and at 3 mg/kg for 21 days, respectively, as single agents or in combination. Upon completion of the gilteritinib or combination schedule (Day 37), tumour volume assessment in the MV‐4‐11 model showed a 57.2% and 84.1% TVI for MEN1703 and gilteritinib, respectively (Figure 5A). One complete response was observed in the gilteritinib group; the combination treatment induced a 99.2% TVI with 5/7 mice in complete response. Complete TVI in the combination group was evident until Day 47, whereas single agents showed tumour regrowth. No relevant body weight changes were observed during treatment (Figure S6A,B). Pharmacodynamic assessment showed a decrease in pFLT3, pSTAT5 and pS6 within the tumour masses that correlated with tumour reduction, demonstrating stronger inhibition with the combination of MEN1703 and gilteritinib (Figure 5B). Synergistic activity was observed at different dose levels of the drugs (Figure S6C–H). Combined treatment resulted in long‐lasting antitumor activity: 6 days to regrowth for 7.5 mg/kg MEN1703 + 3 mg/kg gilteritinib, 14 days to regrowth for 15 mg/kg MEN1703 + 3 mg/kg gilteritinib and 47 days to regrowth for 25 mg/kg MEN1703 + 30 mg/kg gilteritinib (Table S2 and Figure S6C–H). In general, the combination was well tolerated in terms of body weight changes, clinical signs and death events (Table S2 and Figure S6I–N).

**FIGURE 6:**
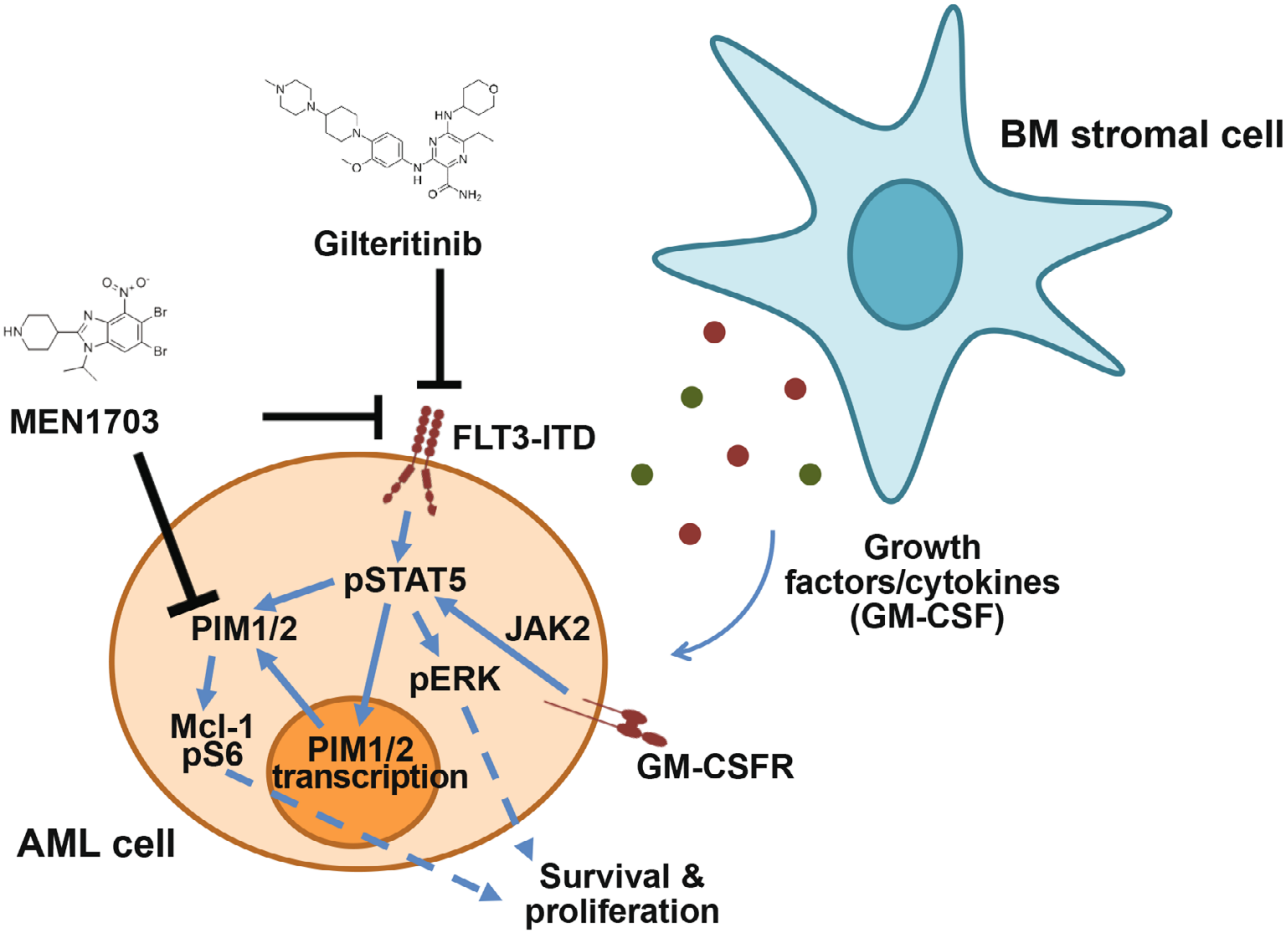
MEN1703 and gilteritinib target distinct pathways whose activation is dependent on the tumour microenvironment. Schematic cartoon representing cytokine‐modulated signalling. FLT3‐ITD stimulates both proliferation and cell survival fostering downstream PIM kinases. Stromal cytokines activate JAK2‐STAT5‐PIM pathway through the GM‐CSF or IL‐3 receptor, boosting the pro‐survival signalling of tumour cells. Inhibition of cell survival may be more effective when both FLT3 and PIM inhibitors simultaneously block the FLT3‐ITD and cytokine signalling. As MEN1703 is a dual FLT3/pan‐PIM inhibitor, its action may strongly reinforce the impairment of proliferation signals that are responsible for resistance to FLT3 inhibitors.

We tested the MEN1703 and gilteritinib combination in a FLT3‐ITD relapse/refractory AML diffuse PDX model (CTG‐2228). MEN1703 and gilteritinib were administered at clinically relevant doses of 15 mg/kg as single agents and in combination, starting concurrently at Day 0. In peripheral blood, the combination treatment produced a statistically significant improvement in antitumor activity versus each agent at Day 48 (by % hCD45 cells, *p* < 0.0001 vs. MEN1703 arm and *p* = 0.0002 vs. gilteritinib arm) (Figure 5C,D and Figure S7A–C). The combination also resulted in a statistically significant reduction in the absolute number and percentage of viable hCD45 blasts in the bone marrow compartment compared to the vehicle and single agents (*p* < 0.0001 vs. vehicle; *p* = 0.0004 vs. MEN1703; *p* = 0.01 vs. gilteritinib) (Figure 5E,F). The tumour reduction was also statistically significant according to the absolute number of hCD33 cells (Figure S7D). In the spleen, the same trend of reduction was observed (Figure S7E). No relevant changes in absolute body weight were recorded (Figure S7F). The lower efficacy of MEN1703 monotherapy on hCD45+ compared to gilteritinib might be explained by weaker inhibition of FLT3 phosphorylation and additional effect of gilteritinib on AXL pathway [23]. However, MEN1703 also suppresses PIM kinases, whose inhibition can delay gilteritinib resistance. This mechanism is likely responsible for the stronger antitumoral efficacy observed in MEN1703 + gilteritinib‐treated animals. Together, combination treatment significantly enhanced single‐agent antitumoral activity in vivo, corroborating the role of the microenvironment in MEN1703's mechanism of action (Figure 6).

## 4 Discussion

Despite the potency of FLT3 inhibitors such as gilteritinib, these agents are frequently inactivated by the emergence of resistance. Cytokines, growth factors and other components of the bone marrow microenvironment, together with several somatic mutations, are responsible for FLT3 inhibitor resistance [11, 12]. Moreover, when FTL3 inhibitors are used as single agents, they result in short‐term responses [5], so development of tolerable and effective combinations is an urgent, unmet need for AML. Pharmacological strategies suggested to overcome such resistance include exploiting the role of PIM kinases in modulating resistance to FLT3 inhibitors [10, 12, 24, 25, 26]. MEN1703 is a first‐in‐class, dual pan‐PIM and FLT3 inhibitor developed to overcome the limitations of selective FLT3‐ITD or PIM inhibitors [9]. In this study, we showed that MEN1703 affects the FLT3 pathway at clinically relevant concentrations, with inhibition of both total and phosphorylated FLT3 protein. This effect contrasts with gilteritinib, which strongly inhibited FLT3 phosphorylation, inducing the upregulation of total FLT3, potentially contributing to gilteritinib inactivation, as previously reported [20].

A positive interaction between MEN1703 and gilteritinib was observed in in vitro experiments in which the drugs exerted synergistic cytotoxicity in both AML cell lines and primary samples. Upon exploring the effects of the single agents and their combination on protein expression levels, we found that pathway inhibition is consistent with a cooperative interaction between MEN1703 and gilteritinib. Indeed, the simultaneous inhibition of pFLT3 by gilteritinib and of PIM downstream modulators Mcl‐1 and pS6 by MEN1703 could be drivers of synergistic interaction. The combination of MEN1703 and gilteritinib decreased these proteins at levels comparable to the single agents, supporting the concurrence between different mechanisms of action of the drugs. Also, the different effect on caspase 3 cleavage seems to confirm the divergent behaviour of the two drugs on the apoptotic pathway.

Studies have demonstrated that early/primary resistance to FLT3 inhibitors is mediated by stromal factors re‐activating the JAK2‐STAT5‐PIM axis and inhibition of these factors could have a positive effect in restoring FLT3 inhibitor efficacy [5, 11, 27]. Interestingly, when we performed differential gene expression and pathway enrichment analysis on MV‐4‐11 cells treated or not with MEN1703, gilteritinib, or the combination, we observed that only MEN1703 or combination treatment inhibited pathways related to the TME.

Through IPA analysis, we observed MEN1703‐mediated negative modulation of many cytokines that play a role in FLT3 inhibitor resistance (including CSF1, IL3, FLT3 ligand, CCL5 and IL6) [22]. Notably, MEN1703 has been previously described to also inhibit ERK signalling, which is activated by bone marrow stroma and responsible for extrinsic resistance to FLT3 inhibitors [9, 28]. In an in vitro model, we demonstrated that treatment with IL‐3 + GM‐CSF or G‐CSF induced significant resistance to gilteritinib. In contrast, in the presence of cytokines, MEN1703 functioned in the same manner as in their absence, both confirming that MEN1703 can affect pathways involved in the TME and echoing results on the role of PIM kinases in cytokine‐mediated rescue of blasts [11]. Indeed, upon repeating the MEN1703‐gilteritinib combination experiments in the presence of cytokines/stromal factors, we found that the drug interaction between MEN1703 and gilteritinib became significantly more synergistic at all dose levels tested, likely due to MEN1703's effect in restoring susceptibility of AML cells to gilteritinib. Comparable results were also achieved in ex vivo experiments with AML FLT3‐ITD cell lines co‐cultured in the presence of primary stromal cells.

Evaluation of the MEN1703‐gilteritinib combination in in vivo FLT3‐ITD AML models further corroborated our in vitro results. In both the MV‐4‐11 xenograft model and a diffuse PDX model of FLT3‐ITD AML, the combination resulted in statistically significant tumour regression at clinically relevant doses of the compounds, with a delay in tumour regrowth at lower concentrations of MEN1703 and gilteritinib and minimal impact on tolerability. Biomarker analysis of tumour masses is quite comparable to in vitro results, but in vivo MEN1703 alone seems to inhibit less STAT5 phosphorylation, with only one animal out of six showing MEN1703‐induced STAT5 decrease. This might be due to a redundant activation of the JAK2/STAT5 axis by TME factors and to the higher heterogeneity of the in vivo setting. Nevertheless, when MEN1703 and gilteritinib cooperate in the inhibition of PIM/FLT3 downstream mediators, strong antileukaemic activity is observed.

## 5 Conclusions

In conclusion, we demonstrated that MEN1703 exerts concurrent inhibition of the PIM pathway and total FLT3 with weaker inhibition of FLT3 phosphorylation. Furthermore, our research indicates that MEN1703 can restore sensitivity to gilteritinib under gilteritinib‐refractory conditions via targeting stromal factor signalling. This observation raises the possibility of using MEN1703 to eliminate minimal residual disease in patients treated with gilteritinib. The effect on TME may explain the increased antitumor activity observed with the combination of MEN1703 and gilteritinib, particularly in the in vivo setting. The in vivo efficacy of the combination, also at dose levels remarkably lower than the standard clinical exposure of single agents, confirms our in vitro results and suggests important translational implications.

## Author Contributions

**Sonia Zicari:** conceptualization (equal), data curation (lead), formal analysis (lead), investigation (lead), methodology (equal), project administration (equal), supervision (equal), writing – original draft (equal), writing – review and editing (lead). **Giuseppe Merlino:** data curation (equal), formal analysis (equal), investigation (equal), writing – review and editing (equal). **Alessandro Paoli:** data curation (equal), formal analysis (equal), investigation (equal), writing – review and editing (equal). **Alessio Fiascarelli:** data curation (equal), formal analysis (equal), investigation (equal), writing – review and editing (equal). **Patrizia Tunici:** data curation (equal), formal analysis (equal), investigation (equal), writing – review and editing (equal). **Diego Bisignano:** data curation (equal), formal analysis (equal), investigation (equal), writing – review and editing (equal). **Francesco Belli:** data curation (equal), formal analysis (equal), investigation (equal), writing – review and editing (equal). **Clelia Irrissuto:** data curation (equal), formal analysis (equal), investigation (equal), writing – review and editing (equal). **Simone Talucci:** data curation (equal), formal analysis (equal), investigation (equal), writing – review and editing (equal). **Elena Cirigliano:** data curation (equal), formal analysis (equal), investigation (equal), writing – review and editing (equal). **Maria Luisa Iannitto:** data curation (equal), formal analysis (equal), investigation (equal), writing – review and editing (equal). **Mario Bigioni:** data curation (equal), formal analysis (equal), investigation (equal), writing – review and editing (equal). **Alessandro Bressan:** data curation (equal), formal analysis (equal), investigation (equal), writing – review and editing (equal). **Krzysztof Brzózka:** data curation (equal), formal analysis (equal), investigation (equal), writing – review and editing (equal). **Gabriel Ghiaur:** conceptualization (supporting), data curation (equal), formal analysis (equal), investigation (equal), methodology (equal), supervision (equal), writing – review and editing (equal). **Daniela Bellarosa:** conceptualization (equal), data curation (lead), formal analysis (lead), investigation (lead), methodology (equal), project administration (equal), supervision (equal), writing – original draft (equal), writing – review and editing (lead). **Monica Binaschi:** conceptualization (equal), data curation (lead), formal analysis (lead), investigation (lead), methodology (equal), supervision (equal), writing – original draft (equal), writing – review and editing (lead).

## Conflicts of Interest

S.Z., G.M., A.P., A.F., P.T., D. Bisignano, F.B., C.I., S.T., E.C., M.B., M.L.I., A.B., D. Bellarosa and M.B. report employment at Menarini/Stemline. K.B. reports employment and CSO at Ryvu Therapeutics; patents, royalties or other intellectual property at Ryvu Therapeutics; stock or stock options at Ryvu Therapeutics, Selvita, Ardigen; advisory board at Ardigen. G.G. reports research funding from Menarini/Stemline, Abbvie, Kinomica, Arcellx; advisory board at Syros; patent US 2020/0345770.

## Supporting information


**Figure S1.** MEN1703 inhibits FLT3 pathway differently from gilteritinib.
**Figure S2.** In vitro combination of MEN1703 and gilteritinib demonstrates a synergistic drug interaction in FLT3‐ITD AML cell lines.
**Figure S3.** MEN1703, gilteritinib and their combination induce mainly inhibition of PIM‐1 mRNA.
**Figure S4.** MEN1703 and drug combination induce cleavage of caspase‐3 differently from gilteritinib.
**Figure S5.** Gilteritinib resistance induced by stromal cytokines in FLT3‐ITD AML cell lines and in primary samples is blocked by MEN1703.00
**Figure S6.** Combination of MEN1703 plus gilteritinib potently inhibits in vivo tumour growth in xenografted FLT3‐ITD AML cell lines.
**Figure S7.** Combination of MEN1703 plus gilteritinib potently inhibits in vivo tumour growth in xenografted FLT3‐ITD AML PDX samples.

**Table S1.** Characteristics of ex vivo samples from AML patients.
**Table S2.** In vivo antitumor activity of MEN1703, gilteriti..nib and the combination in the MV‐4‐11 cell‐derived xenograft model.

**Table S3.** Differentially expressed genes (DEGs) derived from RNA‐Seq.

## Data Availability

Menarini Group will review requests individually to determine whether (1) the requests are legitimate and relevant and meet sound scientific research principles, (2) the requests are within the scope of the participants' informed consent and (3) the request is compliant with any applicable law and regulation and any contractual relationship that Menarini Group and its affiliates and partners have in place with respect to the study and/or the relevant product. Prior to making data available, requestors will be required to agree in writing to certain obligations, including without limitation, compliance with applicable privacy and other laws and regulations. Proposals should be directed to medicalinfo@menarini-stemline.com.
