# Supplementary material for: The Dual PIM/FLT3 Inhibitor MEN1703 Combines Synergistically With Gilteritinib in FLT3‐ITD‐Mutant Acute Myeloid Leukaemia
Source: J Cell Mol Med. 2024 Dec 9;28(23):e70235. doi: 10.1111/jcmm.70235 (PMC11628189; doi:10.1111/jcmm.70235)

Suppl Figure 3

A

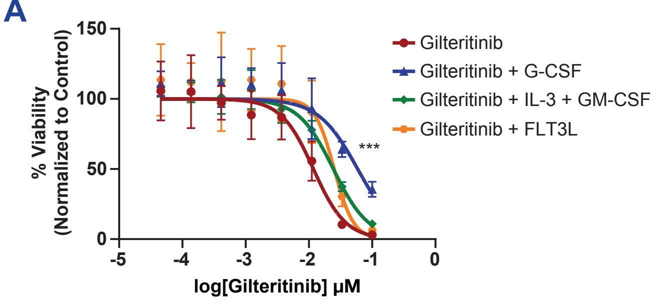

	IC_{50} (µM)	SE
**Gilteritinib**	0.0118	0.0035
**Gilteritinib + G-CSF**	0.0591	0.0178
**Gilteritinib + IL-3 + GM-CSF**	0.0244	0.0057
**Gilteritinib + FLT3L**	0.0252	0.0054

B

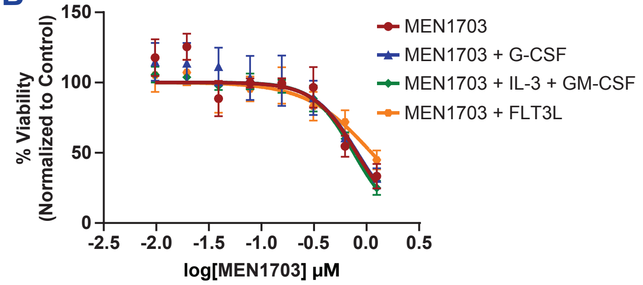

	IC_{50} (µM)	SE
**MEN1703**	0.7999	0.0571
**MEN1703 + G-CSF**	0.8135	0.0525
**MEN1703 + IL-3 + GM-CSF**	0.7520	0.0186
**MEN1703 + FLT3L**	1.1070	0.0609

C

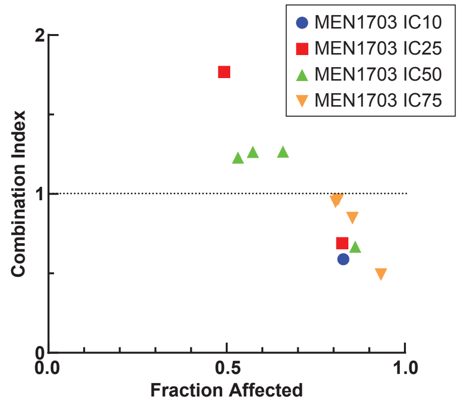

D

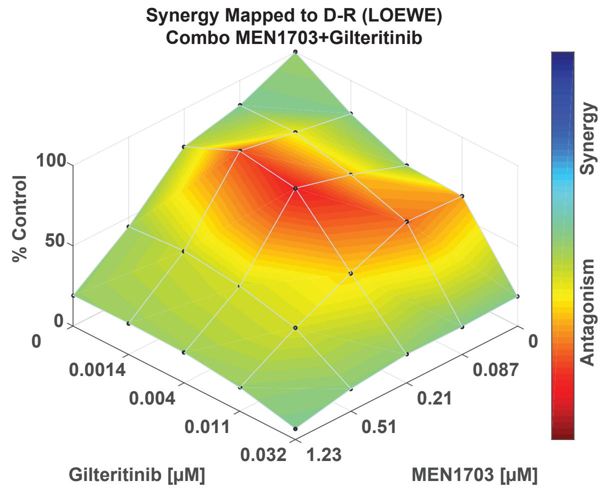

E

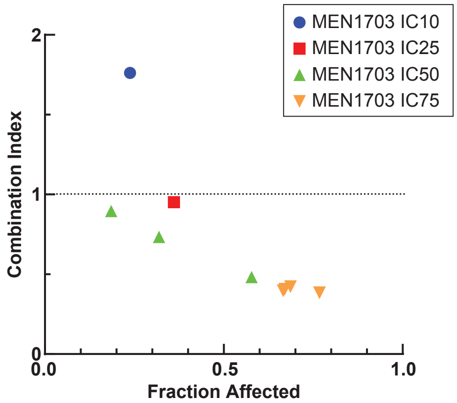

F

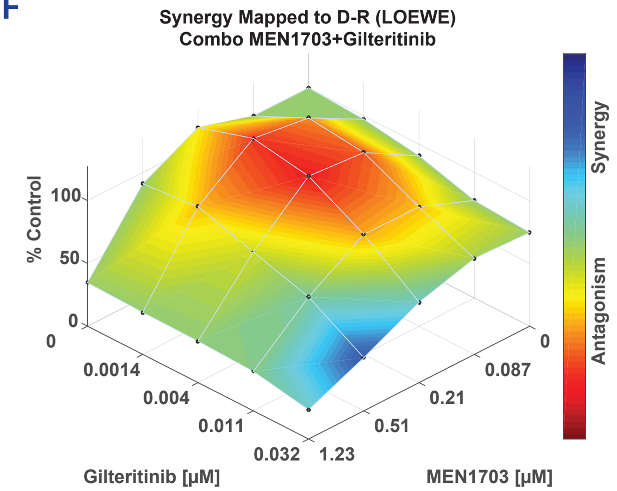

Suppl Figure 3

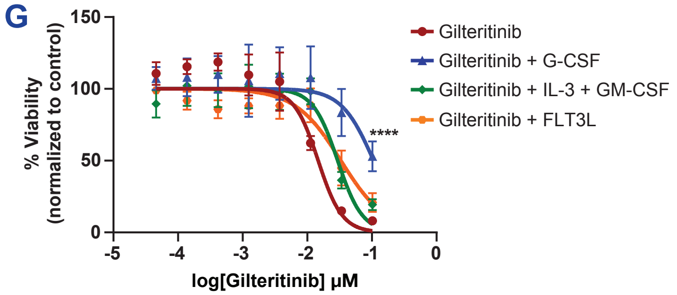

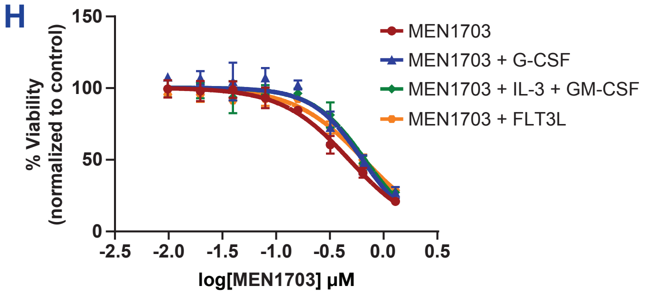

	IC_{50} (μM)	SE
**Gilteritinib**	0.0145	0.0012
**Gilteritinib + G-CSF**	0.1045	0.0143
**Gilteritinib + IL-3 + GM-CSF**	0.0291	0.0497
**Gilteritinib + FLT3L**	0.0326	0.0004

	IC_{50} (μM)	SE
**MEN1703**	0.4659	0.0674
**MEN1703 + G-CSF**	0.6194	0.0468
**MEN1703 + IL-3 + GM-CSF**	0.6531	0.0460
**MEN1703 + FLT3L**	0.6377	0.0583

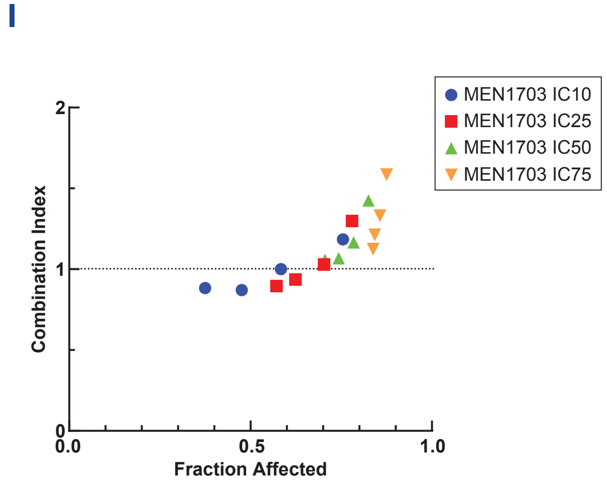

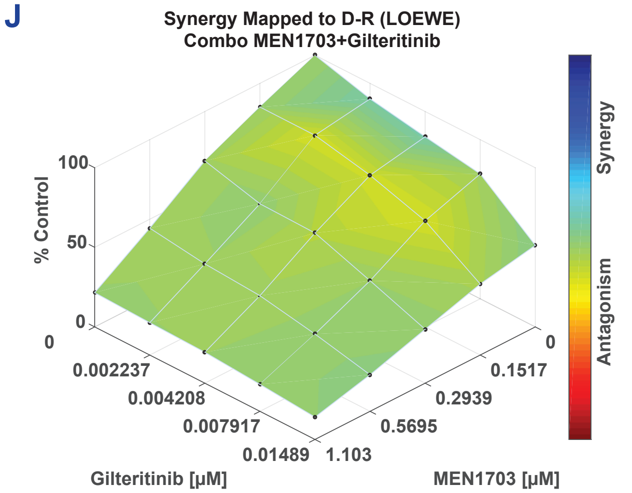

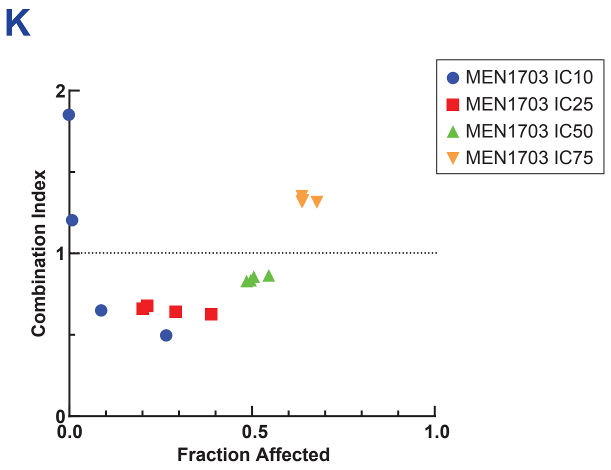

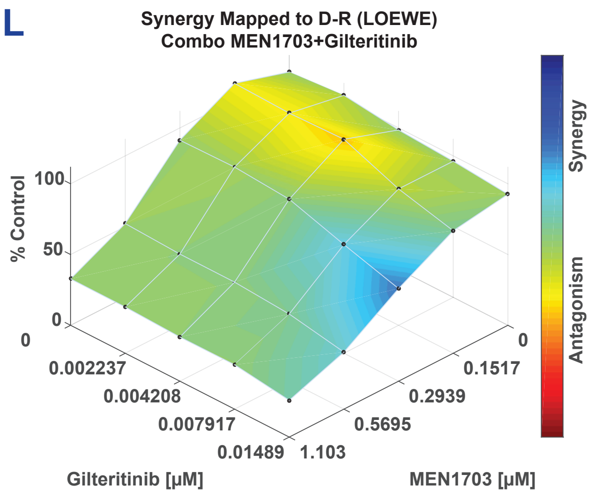

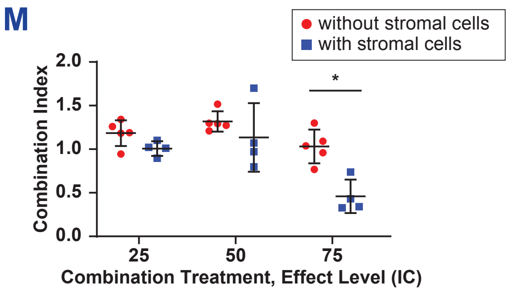

Supplement: Supplementary file 1 — Figure S1. MEN1703 inhibits FLT3 pathway differently from gilteritinib. Figure S2. In vitro combination of MEN1703 and gilteritinib demonstrates a synergistic drug interaction in FLT3‐ITD AML cell lines. Figure S3. MEN1703, gilteritinib and their combination induce mainly inhibition of PIM‐1 mRNA. Figure S4. MEN1703 and drug combination induce cleavage of caspase‐3 differently from gilteritinib. Figure S5. Gilteritinib resistance induced by stromal cytokines in FLT3‐ITD AML cell lines and in primary samples is blocked by MEN1703.00 Figure S6. Combination of MEN1703 plus gilteritinib potently inhibits in vivo tumour growth in xenografted FLT3‐ITD AML cell lines. Figure S7. Combination of MEN1703 plus gilteritinib potently inhibits in vivo tumour growth in xenografted FLT3‐ITD AML PDX samples. [file JCMM-28-e70235-s001.zip › Suppl Figure 5_v3.pdf]
