# Supplementary material for: The Dual PIM/FLT3 Inhibitor MEN1703 Combines Synergistically With Gilteritinib in FLT3‐ITD‐Mutant Acute Myeloid Leukaemia
Source: J Cell Mol Med. 2024 Dec 9;28(23):e70235. doi: 10.1111/jcmm.70235 (PMC11628189; doi:10.1111/jcmm.70235)
Supplement: Supplementary file 2 — Table S1. Characteristics of ex vivo samples from AML patients. Table S2. In vivo antitumor activity of MEN1703, gilteriti..nib and the combination in the MV‐4‐11 cell‐derived xenograft model. [file JCMM-28-e70235-s002.docx]

**Supporting information Table S1.** **Characteristics of ex vivo samples from AML patients**

| Patients | Gene mutation | AA change | % VAF | MEN1703 IC_50_ (µM) | Gilteritinib | Median combination index (CI) | Gilteritinib-treated |
| --- | --- | --- | --- | --- | --- | --- | --- |
| 1 | RUNX1 | p.R204 | 40.76 | 0.30 | 0.0025 | 1.07 | No |
|  | U2AF1 | p.S34F | 35.98 |  |  |  |  |
|  | DNMT3A | p.L754P | 39.79 |  |  |  |  |
|  | FLT3 | ITD, 375bp | 30 |  |  |  |  |
| 2 | FLT3 | ITD, 331bp | 90 | 0.35 | 0.0002 | 0.90 | No |
| 3 | NPM1 | p.W288fs | 25.57 | 0.30 | 0.0011 | 1.14 | No |
|  | DNMT3A | p.R882H | 40.62 |  |  |  |  |
|  | EP300 | p.M1989R | 39.26 |  |  |  |  |
|  | FLT3 | ITD, 381bp | 54 |  |  |  |  |
| 4 | NPM1 | p.W288fs | 20.19 | 0.4 | >10 | 0.95 | Yes |
|  | PTPN11 | p.E76A | 34.99 |  |  |  |  |
|  | FLT3 | p.A680V | 36.7 |  |  |  |  |
|  | EZH2 | p.N315T | 47.07 |  |  |  |  |
|  | KDM2B | p.P787L | 51.28 |  |  |  |  |
|  | KDM6A | p.K220N | 52.5 |  |  |  |  |
| 5 | NPM1 | mutated | NA | 0.24 | 0.23 | 1.20 | Yes |
|  | CEBPa | Mutated | NA |  |  |  |  |
|  | FLT3 | ITD | NA |  |  |  |  |

The table indicates gene mutations for each patient.

AA change (homozygous dominant), %VAF (Variant Allele Frequency), IC_50_, CI (combination index), and TKI (tyrosine kinase inhibitors) treatment were reported.

**Supporting information Table S2**. **In vivo antitumor activity of MEN1703, gilteritinib, and the combination in the MV-4-11 cell-derived xenograft model**

| Compound | Dose (mg/kg) | Schedule | RoA | TVI% Day 37 | Day to CR | Day to regrowth | Death events | Clinical signs |
| --- | --- | --- | --- | --- | --- | --- | --- | --- |
| MEN1703 | 7.5 | Q1xd14 | OS | 41.1 | – |  | 0/7 | – |
| MEN1703 | 15 | Q1xd14 | OS | 44.7 | – |  | 0/7 | – |
| MEN1703 | 25 | Q1xd14 | OS | 72.9 | – |  | 0/7 | – |
| Gilteritinib | 3 | Q1xd21 | OS | 81 | – |  | 0/7 | – |
| Gilteritinib | 30 | Q1xd21 | OS | 100 | 33 | 43 | 0/7 | – |
| Combo 1 (MEN1703 7.5 mg/kg + gilt 3 mg/kg) | – | – | – | 100 | 37 | 6 | 0/7 | – |
| Combo 2 (MEN1703 15 mg/kg + gilt 3 mg/kg) | – | – | – | 100 | 29 | 14 | 0/7 | – |
| Combo 3 MEN1703 25 mg/kg + gilt 30 mg/kg) | – | – | – | 100 | 29 | 47 | 0/7 | Mild to moderate; ruffled hairs, hunching |

The table indicates doses, schedule, oral (OS) route of administration (RoA), % tumor volume inhibition (TVI) evaluated at day 37, days to complete remission (CR), days to tumor regrowth, death events, and clinical signs. (Day to CR: days from the start of treatment to achieve the complete tumor remission or complete response; Day to regrowth: days from complete remission or complete response to tumor regrowth).
